# 4-Aminopyridine treatment for nerve injury resulting from radical retro-pubic prostatectomy: a single-center double-blind, randomized, placebo-controlled study

**DOI:** 10.1186/s13063-024-08102-z

**Published:** 2024-05-21

**Authors:** Ahmed Ghazi, Thomas L. Osinski, Changyong Feng, Andrea Horne, John Elfar

**Affiliations:** 1 Present Address: Urology, Johns Hopkins University, Baltimore, United States; 2 Present Address: University of Rochester, Rochester, USA; 3 Present Address: Orthopaedic Surgery, University of Arizona, Tucson, United States; 4 Orthopaedic Surgery, University of Arizona, Tucson, United States

**Keywords:** Nerve injury, Prostatectomy, 4-Aminopyridine, 4AP, Erectile dysfunction

## Abstract

**Background:**

Prostate cancer (PCa) is the most common non-cutaneous malignancy in men and leads to the second most common cause of cancer related mortality in men. Early detection of PCa allows for a potentially curative intervention. Most men will live over a decade from the time of their PCa diagnosis. Thus, treatments must balance curative interventions with their impact on quality of life. Radical prostatectomy (RP) is one such potentially curative intervention but often leads to erectile dysfunction (ED) and urinary incontinence (UI). Approximately 90,000 RPs are performed each year in the USA. Post-operative ED and UI is thought to occur in part from traumatic peripheral nerve injury (TPNI) to the neurovascular bundles that surround the prostate. Thus, patients undergoing RP may be a population that would benefit from clinical studies that look at TPNI.

**Methods:**

The study is a single-institution, double-blinded placebo-controlled, randomized clinical trial in which patients immediately post-RP receive either 4-aminopyrdine (4AP) or placebo in a 1:1 fashion. The primary outcome is evaluation of the efficacy of 4AP in accelerating the early return of baseline erectile and urinary function post-radical prostatectomy.

**Discussion:**

This study is critical as it could reduce the morbidity associated with RP, a commonly performed operation, and identify a patient population that may greatly benefit into further TPNI research.

**Trial registration:**

ClinicalTrials.gov NCT03701581. Prospectively registered on October 10, 2018.

## Administrative information

Note: The numbers in curly brackets in this protocol refer to the SPIRIT checklist item numbers. The order of the items has been modified to group similar items (see http://www.equator-network.org/reporting-guidelines/spirit-2013-statement-defining-standard-protocol-items-for-clinical-trials/).

**Table:** 

Title {1}	4-Aminopyridine treatment for nerve injury resulting from radical retro-pubic prostatectomy: a single-center double-blind, randomized, placebo-controlled study
Trial registration {2a and 2b}	ClinicalTrials.gov NCT#03701581
Protocol version {3}	Version date 03/18/2024
Funding {4}	Institutional support from the University of Rochester
Author details {5a}	John Elfar, MD, Tenured Professor and Chairman, Orthopaedic Surgery, University of Arizona, Ahmed Ghazi, MD, Associate Professor of Urology, Johns Hopkins University. Thomas Osinski, MD, Assistant Professor, University of Rochester Medical Center, Andrea Horne, CCRP, CCRC, Research Program Administration Manager, Orthopaedic Surgery, University ofArizona. Changyong Feng, PhD, Professor of Biostatistics and Computational Biology, Professor of Anesthesiology, University of Rochester Medical Center.
Name and contact information for the trial sponsor {5b}	Not applicable. This is an investigator-initiated clinical trial.
Role of sponsor {5c}	Not applicable. This is an investigator-initiated clinical trial.

## Introduction

### Background and rationale {6a}

Prostate cancer (PCa) is the most common non-cutaneous cancer in men and the second most common cancer to cause death in men (after lung cancer) worldwide, counting 268,490 new cases and causing 34,500 deaths in the USA alone in 2022. Importantly, most men will live for 10 years or more after a diagnosis of PCa. Most PCa is localized at the time of detection, for which there are several options. For clinically localized disease in healthy men, most are left choosing between radical prostatectomy (RP) and radiation therapy (RT). From an oncologic perspective, surgery and radiation have equivalent outcomes. With similar oncologic outcomes between treatment modalities and long survival from diagnosis, patients are left balancing the risks and benefits of each treatment approach. Despite RP being proven to decrease PCa-specific mortality and progression to metastatic disease, RP is still associated with significant quality of life issues, including worsening of erectile function and urinary continence [1]. RP offers whole-gland pathology analysis with lymph node dissection to give more complete staging and an easier ability to interpret Prostate Specific Antigen (PSA) values in the post-operative setting. However, most men will experience iatrogenic urinary incontinence (UI) and erectile dysfunction (ED) post-operatively that may improve over time [2]. This discussion also underscores the need for improvement in our current treatments for localized PCa.

The technique for RP has been refined over the last two decades and has significantly improved the outcomes. Nerve-sparing RP (NSRP) has been shown to reduce the extent of ED and UI post-operatively. NSRP is performed by dissecting the neurovascular bundles off the posterolateral aspect of the prostate on each side. Occasionally, the nerves cannot be spared due to oncologic concerns or if significant inflammation is present. Even when NSRP is performed, approximately 30–60% of men have postoperative ED 1-year after surgery [3, 4]. UI post-RP is complex, and its causes can be multifactorial; however, a component may be the result of mechanical injury to the urinary sphincter from surgical trauma and/or to the peripheral nerves juxtaposed with the prostate that innervate the external urinary sphincter.

Despite all efforts made during a NSRP, much of the ED and UI experienced is still due to traumatic peripheral nerve injuries (TPNI) [5]. TPNI occurs from traction, thermal, or crush trauma. This affects nerves as well as the distal organ and sensory function. A deeper understanding of TPNI emerged only recently. The degree of nerve damage after NSRP is unknown as there are no intraoperative or post-operative clinical tools to evaluate the extent of nerve damage. Clinically, ED and UI can be measured before and after surgery with validated scales such as the simplified International Index of Erectile Function (IIEF-5) and the Michigan Incontinence Symptom Index (M-ISI) instruments, respectively.

Given this background, RP patients offer a reasonable clinical venue to test for the capacity to respond to TPNI treatment as compared to untreated controls. A key target of inquiry is the loss of erectile function, or potency, and urinary continence which are known to be significantly related to neurovascular injury sustained during RP. Currently, recovery of potency and continence in these patients is inconsistent, unpredictable, and often delayed up to 12 months.

Experiments performed using long-standing standard animal models of acute TPNI reveal novel abilities of 4-aminopyridine (4AP) to enhance both the speed and extent of neurologic recovery. 4AP has been employed in the clinic since the early 1980s as a means of providing symptomatic relief in chronic neurodegenerative disorders including multiple sclerosis, myasthenia gravis, and chronic sequelae of spinal cord injury [6–9]. The major mechanism of action is by inhibiting voltage-activated potassium channels. Those undergoing NSRP offer a unique opportunity to test the viability of 4AP to promote accelerated clinical recovery after TPNI. After TPNI, nerve recovery can occur weeks to years after injury. Clinically, patients begin regaining erectile function and continence as well weeks to years after surgery. Given these factors, we chose a 2-month time course of treatment as this is when the steepest recovery curves occur, thus the best time to evaluate if 4AP is helpful in functional recovery after NSRP.

4AP is currently the mainline treatment in the setting of multiple sclerosis [6, 10] and is used in some of the most fragile of neurologically ailing patients. Multiple sclerosis is a demyelinating disorder that affects the peripheral and central nervous systems. The axonal myelin covering is essential for normal nerve impulse conduction. Crush injuries to nerves can damage myelin sheaths while leaving nerve axons intact. This has led to the idea to study the treatment of peripheral nerve traumatic injuries in humans using 4AP.

The published findings relevant to this proposal are as follows:

1. 4AP administration in a single dose given directly after TPNI leads to a significant reduction in the dysfunction caused by a sciatic nerve crush injury.
2. Daily treatment with 4AP over several weeks leads to accelerated durable recovery over untreated controls. This recovery is maintained after the dose is cleared from the animal and is associated with structural improvements in the injured nerve (enhanced repair of damaged myelin and an increase in the number of nerve fibers (i.e., axons) at the crush site). This finding in particular stands in contradistinction to the effects seen using 4AP for chronic indications, where symptomatic relief is completely dependent on circulating 4AP and cessation of treatment is associated with loss of symptomatic functional benefit.
3. These effects were replicated with a locally implantable form of 4AP, developed in the laboratory, which may allow for targeted dosing in the future.

### Dosing considerations

The routine use of extended-release 4AP at the proposed dose (10 mg BID) is considered safe in humans, and routine testing for the circulating level of 4AP is not typically undertaken. Reference ranges for dose selection are based on clinical experience and the literature from studies establishing the utility of 4AP for other conditions [11]. 4AP has been studied in humans since the early 1980s, and principles of safe usage are well-established [12, 13].

This trial will employ the currently approved slow-release formulation of 4AP to treat erectile dysfunction and incontinence after RRP.

### Objectives {7}

The purpose of this study is to evaluate the role of 4-aminopyridine (4AP) on the course of recovery after peripheral nerve injury specific to that suffered in the setting of RP surgery. The investigational treatment will be used to test the hypothesis that 4AP speeds the often slow and unpredictable recovery after RP.

### Trial design {8}

This is a single-center double-blind, randomized, placebo-controlled, superiority-designed clinical trial to determine if the use of 4AP can accelerate the return of erectile function to baseline levels. The treatment group will receive the active study drug after surgery for 2 months (60 days), and the placebo group will receive a matching placebo after surgery for 2 months (60 days).

## Methods: participants, interventions, and outcomes

### Study setting {9}

This study is conducted in an academic health center. It was approved by the United States Food and Drug Administration. It was approved by the Institutional Review Board at the University of Rochester Medical Center. All patients are obtained from the University of Rochester Medical Center.

### Eligibility criteria {10}

The following are the inclusion criteria:

- Male patients with organ-confined, non-metastatic prostate cancer (stages cT1c–T2c) diagnosed in the last 3 months, planning to undergo robotic-assisted laparoscopic bilateral nerve-sparing radical prostatectomy (NSRP)
- Prostate-specific antigen (PSA) levels less than 15 ng/ml within the last 12 months, with biopsy-proven prostate cancer, for whom postoperative adjuvant therapy (e.g., radiation or androgen deprivation therapy) is not expected to be needed
- Ages 45–75
- An Abridged International Index of Erectile Function-Erectile Function (IIEF-5) score of greater than or equal to 17 at the time of screening
- Has experienced at least 6 months of regular sexual activity and sexual activity during the 12 weeks prior to prostate biopsy or surgery
- Willingness to abstain from treatments for erectile dysfunction until 3 months after surgery
- Willingness to participate and able to provide informed consent

The following are the exclusion criteria:

- Planned adjuvant therapy after NSRP based on specimen pathology and patient preference within the first 6 months from surgery
- Neo-adjuvant therapy prior to NSRP
- History of recurrent prostate cancer
- History of seizures, multiple sclerosis, stroke, or any other diagnosed neurological disorder
- History of non-organ confined or metastatic prostate cancer (clinical stages T3 or greater)
- History of known hypersensitivity to 4AP
- Patients with a history of penile surgery other than circumcision or endoscopic urethral stricture surgery
- Renal impairment based on calculated GFR (GFR < 60 mL/min)
- Use of any other aminopyridine medications for any other indication

### Who will take informed consent? {26a}

Urologists will introduce and discuss the nature of the study and answer any questions the patient may have during an in-person pre-operative visit. To minimize the opportunity for any perceived coercion by the subject’s surgeon, patients expressing interest will be referred to the study coordinator to further the study discussion and obtain informed consent. The study coordinator will obtain informed consent prior to the subject’s surgery.

### Additional consent provisions for collection and use of participant data and biological specimens {26b}

Not applicable. There are no additional consent provisions for this study.

### Interventions

#### Explanation for the choice of comparators {6b}

Our treatment comparator is a placebo because there is no current effective therapy for reversing the effects of nerve injury after NSRP. While PDE5 inhibitors have been beneficial in helping men achieve erections after NSRP, studies have not shown that PDE5 inhibitors improve organic erectile function in men who take PDE5 inhibitors versus those who have not after prostatectomy. Furthermore, we standardize the use of PDE5 inhibitors during the first 3 months after surgery because varied use of PDE5 inhibitors between the groups may create a confounding variable that would be difficult to sort out with a study of this size.

#### Intervention description {11a}

Subjects will be randomized equally into one of two groups. Both the subject and investigators will be blinded to group assignment.

- The treatment group (40 subjects) will receive the study drug daily, in two oral doses, taken at least 12 h apart (approximately 8:00 am; 8:00 pm), starting on the day after surgery with the approximately 8:00 am dose. The treatment group will continue at this same dose and daily dosing regimen for 2 months (60 days).
- The placebo group (40 subjects) will receive a placebo daily, in two oral doses, taken at least 12 h apart (approximately 8:00 am; 8:00 pm), starting on the day after surgery with the approximately 8:00 am dose. The placebo group will continue at this same dose and daily dosing regimen for 2 months (60 days).

The following are the dosing instructions:

- Dosing schedule = 1 capsule in the morning and another capsule 12 h later (approximately 8:00 am; 8:00 pm).
- Dosing to begin the day after surgery with the 8:00 am dose.
- Subjects will not take more than two capsules in a 24-h period.
- Subjects will take the capsules whole. They will not break, crush, chew, or dissolve capsules before swallowing. The subjects will be told that the medication is released slowly over time, and if the tablet is broken, the medicine may be released too fast which can raise the chance of having a seizure.
- The study drug can be taken with or without food.
- If a dose is missed, they should not make up the missed dose; however, subjects may take the dose within 1 h of the scheduled time. They will be told not to take two doses at the same time but to take the next dose at the regular scheduled time.
- If the subject is unable to take the study medication due to surgical complications (such as ileus), the doses will be skipped and the subject will resume the study medication once able to take other medications by mouth. The subject will not make up the missed doses.
- Subjects will be reminded not to take the study drug together with other aminopyridine medications, including compounded 4AP (sometimes called 4-aminopyridine or fampridine).

#### Criteria for discontinuing or modifying allocated interventions {11b}

Subjects will be withdrawn from the study for the following:

- If the subject is unable or unwilling to take the study drug.
- Subjects planned for a NSRP but who did not receive a nerve-sparing procedure intraoperatively.
- Subjects who have experienced a seizure while taking the study medication.
- Subjects who take ED treatments within the 3-month period after surgery.
- Any subject with an eGFR less than 60 mL/min/1.73 m^2^ while on the study, if clinically indicated, will have a repeat serum BUN and creatinine within 2–3 days, and if eGFR is still less than 60 mL/min/1.73 m^2^, they will be promptly withdrawn from the study.
- In subjects who incidentally demonstrate a decrease in eGFR > 30 mL/min on study, but still maintain an eGFR ≥ 60 mL/min, we will obtain repeat BUN and creatinine within 2–3 days, and if eGFR based on repeat labs is < 60 mL/min, they will be promptly withdrawn from the study.
- Subjects with final surgical pathology showing pT3 disease, positive nodes, or positive surgical margins who require adjuvant treatment (such as androgen deprivation therapy or radiation) as adjuvant treatments can worsen ED and UI after NSRP.
- The Data Safety Monitoring Committee (DSMC) and/or principal investigator may withdraw any subject at increased risk using the best medical opinion based on reported AEs/SAEs.

#### Strategies to improve adherence to interventions {11c}

Subjects will be instructed to complete a drug diary during the treatment period. The diary will be reviewed in all follow-up interactions with the subject. The subject will be asked to return any unused medication at the end of the treatment period to monitor compliance with the study protocol.

#### Relevant concomitant care permitted or prohibited during the trial {11d}

Phosphodiesterase type 5 (PDE5) inhibitors are excluded within the first 3 months of enrollment. Subjects will be screened prior to enrollment to determine previous use of PDE5 inhibitors. Subjects who have taken PDE5 inhibitors more than once a week, because they cannot achieve an erection sufficient for intercourse without the medication, will not be excluded from the study but will be required to discontinue their use for 3 months while enrolled in the study.

#### Provisions for post-trial care {30}

The subject and/or the subject’s insurance company will be responsible for all standard of care costs associated with post-operative radical retro-pubic prostatectomy (PDE5 inhibitors, vacuum pumps, urethral suppositories, injection therapy). Subjects will be encouraged to contact their insurance company for insurance coverage costs prior to the standard of care treatments offered in this study.

No compensation is planned for trial-related injury.

### Outcomes {12}

#### Primary outcome

The primary outcome is return to baseline confidence in the ability to achieve and maintain erection as measured by item 1 of the Abridged International Index of Erectile Function (IIEF-5) by 12 weeks after surgery.

#### Secondary outcomes

The following are the secondary outcomes:

- Return to baseline erectile function as measured by the IIEF-5
- A more rapid recovery (time frame) of subject-reported erectile function measured by the Abridged International Index of Erectile Function (IIEF-5)
- Improved recovery of baseline subject reported continence using the Michigan Incontinence Symptom Index (M-ISI) in the intervention group
- Decreased use of medications and devices for erectile dysfunction measured by the Attempted Sexual Intercourse and Use of Erectile Aids questionnaire

#### Participant timeline {13}


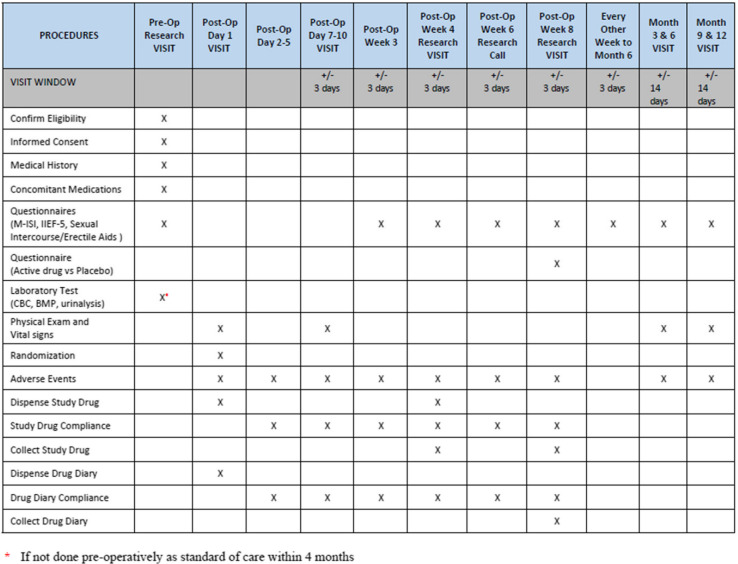


## Research visit 1 (pre-op)

At research visit 1, informed consent will be obtained, eligibility confirmed, medical history and concomitant medications collected, and we will obtain a urinalysis, CBC, and BMP, if not done as pre-operative standard of care.

In addition, two questionnaires will be administered pre-operatively as part of the standard of care practice. The Michigan Incontinence Symptom Index (M-ISI) and the Abridged International Index of Erectile Function (IIEF-5) are standardized measures of the consequences of radical retro-pubic prostatectomy and will be used to track this debilitating set of consequences. Subjects will also complete a 3rd questionnaire, the Attempted Sexual Intercourse and Erectile Aids questionnaire, as a part of this study.

## Post-op day 1

Subjects will have a visit with study personnel on the first day after their surgical procedure.

Greater than 90% of those who undergo robot-assisted RRP are discharged on post-operative day #1. A physical exam and vital signs will be obtained as part of the standard of care. Subjects will begin treatment (either study drug or placebo) while in the hospital, prior to discharge. This will allow dosing instructions to be clearly reviewed with subjects and the first dose administered while in the care of a facility. Initial dose time and date will be logged in the subject drug diary with the subject and accompanying family members to help emphasize the adherence to the protocol during the 2-month treatment period. Dosing instructions, study drug compliance, and completion of the subject drug diary will be reviewed in all follow-up interactions with the subject. Subjects will be instructed to return bottle #1 at the week #4 visit.

## Post-op days 2–5

For the first 5 days after surgery, subjects will be contacted by study personnel in person (post-op day #1) and by telephone (post-op days #2–5) to monitor for potential side effects. Any adverse events will be documented. In addition, the study drug and diary will be reviewed to ensure accuracy and completion, and any questions the subjects may have about the study will be answered.

## Post-op days 7–10

On post-op days 7–10 (± 3 days to accommodate provider/subject schedules), subjects will have a standard-of-care clinic visit which will include a brief physical exam (surgical site, abdominal and genital exam, and review of systems) and have vital signs checked. As part of the standard of care, subjects will be encouraged to perform Kegel exercises at this visit.

## Post-op week 3

At post-op week 3, any adverse events will be checked, and the study drug and diary will be reviewed via telephone using the Telephone Log and Script Form. Subjects will complete the three questionnaires (research procedures).

## Post-op weeks 4, 6, and 8

Subjects will have a research visit with study personnel at weeks 4 and 8 (± 3 days), which will include administration of the three questionnaires, study drug and diary compliance, and a check of any adverse events. At post-op week 4, subjects will return bottle #1 of the study drug, and bottle #2 will be dispensed. They will be instructed to return their completed drug diary at each study visit and any unused medication at the end of the treatment period (week 8). As an internal control, subjects will be asked to complete an additional questionnaire at week 8 to assess whether they think they received the active drug or placebo. On week 6, subjects will receive a call from study personnel to assess the adverse events and instruct subjects to complete their questionnaires.

## Post-op week 10 to month 6

Completion of the three questionnaires will continue every other week from post-op week 10 to month 6 (research procedure), and every effort will be made to collect them. Adverse events will be captured and monitored by the study staff.

## Post-op months 3, 6, 9, and 12 visits

As part of the post-operative standard of care, subjects will be seen at 3 months, 6 months, 9 months, and 12 months (± 14 days). Subjects will be offered treatment for erectile dysfunction starting at 3 months post-op and for the duration of the study. Treatment options will be guided by the results from the IIEF-5 questionnaire and algorithm for erectile dysfunction and include oral agents (PDE5 inhibitors) or any type of non-surgical erectile dysfunction treatments (penile pump, urethral suppositories, intracavernosal injections). At these visits, we will obtain the three questionnaires, physical exam, and vital signs (as part of the standard of care) and also query for adverse events. PDE5 inhibitors include drugs such as Viagra (sildenafil), Cialis (tadalafil), and Levitra (vardenafil) will be the drugs prescribed for post-operative erectile dysfunction as part of the standard of care. The surgeon and/or a member of the study team will be the prescriber of these medications 3 months after surgery. We will review PDE5 use with post-operative questionnaires.

Subject medical records will be reviewed to collect basic demographic information, as well as information about the subject’s medical and surgical history, diagnosis, surgery, clinical lab and pathology results, imaging results, and clinical outcomes.

## Sample size {14}

The primary outcome is return to baseline ability to achieve and maintain erection as measured by Item of the Abridged International Index of Erectile Function (IIEF-5) by 12 weeks after surgery. Suppose at the conclusion of the follow-up period, the rates of returning to the baseline ability in the treatment and control groups are 70% and 30%, respectively. Pearson’s chi-square test indicates that a sample size of 56 (with 28 individuals in each group) achieves 90% power to detect the intended difference of 40% (at a significance level of 0.05). Accounting for an estimated dropout rate of 30%, we plan to recruit 40 subjects in each group.

## Recruitment {15}

The inclusion age range of subjects has been set at 45–75 which reflects the subject age demographic and time of disease presentation with consideration for surgical intervention for prostate cancer at the University of Rochester (URMC) Cancer Center.

### Assignment of interventions: allocation

#### Sequence generation {16a}

Participants are enrolled in this study in a series of four blocks to ensure that there is not a significant skewing of participants receiving the study drug or placebo at any given point. Each block is a series of randomization numbers and then randomly assigned to an active or placebo treatment course. The patient is enrolled during the surgery after the nerve-sparing portion of the surgery, if a sufficient bilateral NSRP was able to be performed. At this time, the surgical team contacts the pharmacy, and the participant is then assigned a computer-generated randomization number. Each randomization number is associated with the date, study subject initials, the study assigned subject number, whether the act of drug or placebo was given, the lot number of the product, the expiration date of the product, and the initials of the pharmacist.

#### Concealment mechanism {16b}

Each prescription is filled with labels that state the drug as “Dalfampridine 10 mg/placebo study drug.” All capsules are packaged by the University Pharmacy using identical size and color capsules. A sealed study subject-specific envelope is created for each subject enrolled that contains an index card that details whether that subject received active or placebo capsules. If the blinded staff is required to unblind themselves, the envelope is opened and the person, date/time of unblinding, and the reason for unblinding are documented.

#### Implementation {16c}

The Investigational Drug Service (IDS) at the University of Rochester will randomize subjects prior to scheduled nerve-sparing radical retro-pubic prostatectomy surgery in a 1:1 ration to the treatment or placebo group.

### Assignment of interventions: blinding

#### Who will be blinded {17a}

Both the subject and the provider will be blinded to group assignment to avoid selection bias. The Investigational Drug Service (IDS) will randomize subjects and dispense study medication and therefore, remain unblinded.

#### Procedure for unblinding if needed {17b}

In the event of a medical emergency, the principal investigator and/or DSMC may decide to disclose drug assignment. The Investigational Drug Service will provide a sealed envelope with the subject’s drug assignment to the blinded study team. If unblinded, the subject will receive no further study drug doses and will be withdrawn from the study.

### Data collection and management

#### Plans for assessment and collection of outcomes {18a}

Standard of care laboratory tests include urinalysis, CBC, and BMP, which will be completed pre-operatively.

Three questionnaires will be administered pre-operatively and post-op week 3. Completion of the three questionnaires will continue every other week from post-op week 4 to month 6 and then again at months 9 and 12. Subjects will complete the questionnaires throughout the study by entering their responses directly into a REDCap database, during an in-person visit, and/or via telephone with the study personnel. REDCap is a secure research data capture program supported by the University of Rochester.

At 3 months post-op, and for the duration of the study, subjects will be offered treatment for erectile dysfunction. Treatment options will be guided by the results from the IIEF-5 questionnaire and algorithm for erectile dysfunction.

As an internal control, subjects will be asked to complete an additional questionnaire at week 8 to assess whether they believe they received the active drug or placebo.

#### Plans to promote participant retention and complete follow-up {18b}

All patients included in the study are on an intention-to-treat basis. Patients who do not complete the full study will have their data included for statistical use. The final outcomes are computed based on these accepted statistical protocols and reviewed by a qualified statistician.

#### Data management {19}

Study data will be collected and maintained in the University of Rochester’s REDCap system. REDCap servers are secured in a local data center at the University of Rochester, and all web-based information transmission is encrypted.

#### Confidentiality {27}

Data will be stored on password-protected and encrypted computers using a secure drive at the University of Rochester Medical Center. Access will be restricted to the investigators, the study coordinator, and any agency legally empowered to demand access to the data.

Subject data is coded using a unique number, and all identifying information (i.e., name, address, phone number) will be stored separately via a study master key linked to the code. The master key will be destroyed by the investigative team after the study is completed and all data has been analyzed.

The results of this study will be published at scientific meetings and in publications without disclosure of subject identity.

#### Plans for collection, laboratory evaluation, and storage of biological specimens for genetic or molecular analysis in this trial/future use {33}

Subject information collected as part of this study will not be distributed or used for future research studies.

## Statistical methods

### Statistical methods for primary and secondary outcomes {20a}

#### Planned statistical analysis

The data analysis will be based on the principle of intention to treat.

(1) Descriptive statistics:

Descriptive statistics for all covariates and outcomes will be reported. For categorical variables, the frequency will be reported. Pearson’s chi-square test or Fisher’s exact test will be used to compare the difference between the two groups. For continuous variables, the mean, standard deviation, and median will be calculated. Two-sample *t*-test (for normally distributed data) or Wilcoxon rank test will be used to compare them in the two groups.

(2) Analysis of outcomes:

(2.1) Analysis of the primary outcome.

- Pearson’s chi-square test will be used to compare the rates of returning to the baseline ability by the end of the follow-up.
- Since this is a longitudinal study, we anticipate some patients will drop out during the follow-up. We assume that the dropout is monotone and random, which means that once they drop out, they will not come back to the study, and the dropout only depends on the information up to the point of dropout. We will model the probability of dropout by logistic regression. The weighted generalized estimation equation (GEE) (with logit link) will be used to study the treatment effects on the returning to the baseline ability at each visit after adjusting the effects of some baseline demographic and clinical factors [14, 15].

(2.2) Analysis of secondary outcomes:

- Return to baseline erectile function as measured by the IIEF-5: The same method as in the primary outcome will be used.
- A more rapid recovery (time frame) of subject-reported erectile function measured by the Abridged International Index of Erectile Function (IIEF-5): The Kaplan–Meier estimator will be used to estimate the distribution function of the time of recovery. The log-rank test will be used to compare the distributions of the two groups.
- Improved recovery of baseline subject reported continence using the Michigan Incontinence Symptom Index (M-ISI) in the intervention group: The same method as in the primary outcome will be used.
- Decreased use of medications and devices for erectile dysfunction measured by the Attempted Sexual Intercourse and Use of Erectile Aids questionnaire: The weighted GEE (with identity link) will be used to study the effects of treatment on the use of medications and devices at each visit after adjusting the effects of some baseline demographic and clinical factors [14].

#### Interim analyses {21b}

Not applicable. No interim analysis is planned.

#### Methods for additional analyses (e.g., subgroup analyses) {20b}

Not applicable. No subgroup analyses are planned.

#### Methods in analysis to handle protocol non-adherence and any statistical methods to handle missing data {20c}

This is a typical longitudinal study. Missing data is almost unavoidable. Although we can never test the missing mechanism assumption based on the observed data, the data missing at random (MAR) is usually a reasonable assumption. In the data analysis, we will make this assumption. The current semiparametric method can only handle the missing data with a monotone missing pattern which means that if the patient misses one scheduled visit, then they will miss all visits thereafter. We will artificially truncate data after the first missed appointment if this occurs.

#### Plans to give access to the full protocol, participant-level data, and statistical code {31c}

The full protocol is published as part of our approved IND at ClinicalTrials.gov. Participant-level data will be reported at ClinicalTrials.gov following the completion of the trial.

### Oversight and monitoring

#### Composition of the coordinating center and trial steering committee {5d}

This trial is overseen by a committee of data safety monitors. The roles of individuals within this committee are equal. Data management in this trial is overseen by the University of Rochester Wilmot Cancer Center Data Management team. This trial is also overseen by the Institutional Review Board at the University of Rochester Medical Center, as per the guidelines outlined in the Investigational New Drug approval obtained by the study team.

#### Composition of the data monitoring committee, its role, and reporting structure {21a}

Study investigators will conduct a continuous review of data and subject safety. The investigators will submit annual progress reports of these data to the Wilmot Cancer Center’s Data Safety Monitoring Committee (DSMC) for review. The review will include the number of subjects enrolled, withdrawals, serious adverse events, both expected and unexpected, and responses observed.

The Data Safety Monitoring Committee (DSMC) consists of Barbara Asselin, MD; Jane Liesveld, MD; Megan Baumgart, MD; Yuhchyau Chen, MD, PhD; KC Clevenger, PhD; Luke Peppone, PhD, MPH; Derick Peterson, PhD; Kyle Richards, PharmD; Michael Cummings, MD; Andrew McDavid, PhD; Andrew Goodman, MD; Edward Messing, MD; and Mark Noble, MD.

#### Adverse event reporting and harms {22}

Adverse events will be tabulated by treatment, severity, and perceived relationship to the study drug.

For each adverse event, comparisons will be performed among 4AP and placebo regarding the occurrence of at least one event using Prescott’s test [15]. Particular attention will be paid to AEs known to be associated with 4AP in previous studies and to serious AEs.

#### Frequency and plans for auditing trial conduct {23}

The procedures for auditing trials conducted at the University of Rochester include yearly, as well as interspersed, sporadic audits conducted by the University of Rochester Institutional Review Board-governed by a trial audit team. This process is independent from the investigators and the sponsor.

#### Plans for communicating important protocol amendments to relevant parties (e.g., trial participants, ethical committees) {25}

All protocol amendments are reviewed by the URMC IRB. Any amendments that would alter a participant’s eligibility or study protocol would be reported directly.

#### Dissemination plans {31a}

The authors intend to collaborate and disseminate the results of this study via publication in the peer review literature. Deidentified results databases will be attached to the publication for independent review.

## Discussion

This study is critical as it provides an important opportunity to look at TPNI in subjects undergoing NSRP. Currently, clinical research on TPNI is challenging, as many who present with TPNI have great variability in the injuries sustained. TPNI can be debilitating, and currently, there are no proven therapies to help recover function. RP is the standard surgical treatment for localized PCa, which includes surgical removal of the prostate while preserving the neurovascular bundle on the posterolateral aspects of the prostate. The surgical dissection performed using a standard approach, although resulting in consistent tension, results in limited variability with postoperative ED and UI. Thus, this procedure would be ideal to investigate a drug that may enhance recovery of controlled TPNI.

Critically, we are limiting the use of PDE5 inhibitors for 3 months after surgery. Often, men have significant ED 3-months after surgery. While PDE5 inhibitors have been shown to improve the ability to achieve erections after RP, they have not been shown to help improve recovery of erectile function. Limiting the use of PDE5 inhibitors for 3 months prevents confounding that would be difficult to interpret without much larger studies. This is an important consideration for those creating trials looking at TPNI in men who undergo NSRP. However, if this study is successful, 4AP would likely become the standard of care and future studies would need to look at whether immediate use of PDE5 inhibitors help enhance recovery of erectile function when taken in combination with 4AP.

This study design will hopefully provide a model to help advance the study of TPNI. Men undergoing NSRP, as well as many others who sustain TPNI from other causes, could benefit from identifying clinically useful medications that help with nerve regeneration after injury.

## Trial status

The currently approved protocol is version date 05/03/2023. Enrollment for this trial began in December 2019. Recruitment is estimated to be completed by mid-2023.

## Data Availability

Deidentified data associated with this trial will be openly available to investigators at the conclusion of the analysis.

## Abbreviations

4AP: 4-Aminopyridine
TPNI: Traumatic peripheral nerve injury
URMC: University of Rochester Medical Center
RRP: Radical retro-pubic prostatectomy
MS: Multiple sclerosis
IR: Immediate release
FDA: Food and Drug Administration
CPK: Creatinine phosphokinase
NSRP: Nerve-sparing radical prostatectomy
PSA: Prostate-specific antigen
IIEF 5: International Index of Erectile Function
ED: Erectile dysfunction
DSMC: Data Safety Monitoring Committee
AE: Adverse event
SAE: Serious adverse event
PDE5: Phosphodiesterase type 5
M-ISI: Michigan Incontinence Symptom Index
IDS: Investigational Drug Service
MAR: Missing at random
GEE: Generalized estimation equation
GFR: Glomerular filtration rate
CBC: Complete blood count
BMP: Basic metabolic panel
