# Correction: 4-Aminopyridine treatment for nerve injury resulting from radical retro-pubic prostatectomy: a single-center double-blind, randomized, placebo-controlled study

**DOI:** 10.1186/s13063-024-08518-7

**Published:** 2024-10-02

**Authors:** Ahmed Ghazi, Thomas L. Osinski, Changyong Feng, Andrea Horne, John Elfar

**Affiliations:** 1 Present Address: Urology, Johns Hopkins University, Baltimore, USA; 2 Present Address: University of Rochester, Rochester, USA; 3 Present Address: Orthopaedic Surgery, University of Arizona, Tucson, USA; 4 Orthopaedic Surgery, University of Arizona, Tucson, USA

**Correction** **:** **Trials 25, 332 (2024)**

**https://doi.org/10.1186/s13063-024-08102-z**

Following the publication of the original article [1], we were notified that the authors would like to include the following disclosure statement:

“Dr. John Elfar holds a patent on 4-aminopyridine and has invested in startup company which intends to manufacture a pill alternative to the currently generic available pill utilized in John Elfar’s sponsored trials. Dr. Elfar served as the Chief Medical Officer at Solaxa (Solaxa.com) until October 2023 and now only serves as a founder.”
